# Parity, lactation, and long‐term weight change in Mexican women

**DOI:** 10.1111/mcn.12988

**Published:** 2020-03-24

**Authors:** Mónica Mazariegos, Eduardo Ortiz‐Panozo, Teresita González de Cosío, Martín Lajous, Ruy López‐Ridaura

**Affiliations:** ^1^ Nutrition and Health Research Center National Institute of Public Health Cuernavaca Mexico; ^2^ Population Health Research Center National Institute of Public Health Cuernavaca Mexico; ^3^ Health Department Iberoamerican University Mexico Mexico; ^4^ Department of Global Health and Population Harvard T.H. Chan School of Public Health Boston Massachusetts

**Keywords:** breastfeeding, lactation, parity, weight gain

## Abstract

One post‐partum behaviour that may be protective against post‐partum weight retention and long‐term weight gain among women of reproductive age is lactation because of its potential role in resetting maternal metabolism after pregnancy. However, most of the evidence focuses on weight retention at 6, 12, or 24 months post‐partum, and data beyond 2 years after birth are sparse, and findings are inconclusive. Therefore, our aim was to assess the association of parity and mean duration of lactation per child with long‐term weight change in Mexican women. We assessed the association of parity and mean duration of lactation per child with long‐term weight change in 75,421 women from the Mexican Teachers' Cohort. Several multivariable regression models were fit to assess these associations. We also examined the non‐linear association between duration of lactation and weight change using restricted cubic splines. We found that parous women (≥4 children) gained 2.81 kg more (95% CI [2.52, 3.10]) than did nulliparous women. The association between mean duration of lactation per child and weight change appeared to be non‐linear. Women who breastfed on average 3–6 months per child had lower gain weight (−1.10, 95% CI [−1.58, −0.47 kg]) than had women who did not breastfeed. This association was linear up to 6 months of lactation per child. Our findings suggest that parity alters weight‐gain trajectory in women and that lactation could reduce this alteration. These findings are important in the prevention of excessive weight gain through reproductive years and their future health implications.

**Key Messages**

- Parity alters weight‐gain trajectory in women of reproductive years, and lactation could reduce this alteration. These findings are important in the prevention of excessive weight gain through reproductive years and their future health implications.
- The association between the mean duration of lactation per child and weight change was linear up to 6 months of lactation per child, after which the less weight gain reached a plateau.
- The non‐linear association between the mean duration of lactation per child and weight change could potentially be explained by the fact that after 6 months of age, the frequency and intensity of lactation decrease as complementary foods are introduced.

**Abbreviations**
BMI: body mass index
FFQ: food frequency questionnaire
MTC: Mexican Teachers' Cohort
INSP (for its Spanish name): National Institute of Public Health of Mexico
SAS: Statistical Analysis System software package
SES: socio‐economic status

## 1 INTRODUCTION

Women are at increased risk of obesity in comparison with men (Poston et al., 2016). Reproductive factors, such as earlier age at menarche and at first pregnancy, higher number of pregnancies, higher gestational weight gain and post‐partum weight retention, limited breastfeeding, and menopause weight changes may lead to higher trajectories of weight gain over women's life (Gunderson, 2009; Poston et al., 2016). Pregnancy‐related weight gain has emerged as a potential cause of excess adiposity and long‐term maternal weight gain independent of age‐related weight gain (Gunderson, 2009; Gunderson, Abrams, & Selvin, 2001).

Current estimates suggest that by 2025, more than 21% of women of reproductive age in the world will be obese (NCD Risk Factor Collaboration Group, 2016). In Latin America, the rate at which obesity prevalence is increasing has been faster than that observed in developed countries (NCD Risk Factor Collaboration Group, 2016).

Many studies have found that weight increases after each birth (Gunderson et al., 2004; Lewis et al., 2000; Rosenberg et al., 2003; Weng, Bastian, Taylor, Moser, & Ostbye, 2004). One post‐partum behaviour that may be protective against post‐partum weight retention and long‐term weight gain among women of reproductive age is lactation because of its potential role in resetting maternal metabolism after pregnancy (Stuebe & Rich‐Edwards, 2009). However, most of the evidence focuses on weight retention at 6, 12, or 24 months post‐partum (He et al., 2015; Neville, McKinley, Holmes, Spence, & Woodside, 2014), and data beyond 2 years after birth are sparse, and findings are inconclusive. Up to now, to the best of our knowledge, just six articles have studied the association between lactation and long‐term weight change (Bobrow, Quigley, Green, Reeves, & Beral, 2013; Kirkegaard et al., 2014, 2015; Palmer, Kipping‐Ruane, Wise, Yu, & Rosenberg, 2015; Sharma, Dee, & Harden, 2014; Sichieri, Field, Rich‐Edwards, & Willett, 2003). Furthermore, it would be relevant to generate evidence to protect, promote, and support breastfeeding in low‐income and middle‐income income countries where breastfeeding practices are suboptimal—and are far from the World Health Organization's recommendation to exclusively breastfeed infants for the child's first 6 months to achieve optimal growth, development, and health—having implications on future health outcomes of infants and women (Victora et al., 2016).

Evidence evaluating the role of lactation on maternal weight change is of critical public health importance and will provide insights on the type of interventions that could be effective in reducing cumulative weight gain throughout reproductive cycles and tackle the burden of maternal obesity‐related illness. Therefore, the aim of this study was to assess the association of parity and mean duration of lactation per child with long‐term weight change in Mexican women.

## 2 METHODS

### 2.1 Study population

This study was based on the Mexican Teacher's Cohort (MTC), described elsewhere (Lajous et al., 2015). Briefly, MTC is an ongoing prospective cohort of female teachers of 25 years and older from 12 states of Mexico enrolled in 2006–2008. Women who responded to the baseline questionnaire in 2008 reported full reproductive history and had no other pregnancies during the 2011 follow‐up were eligible for this study. At baseline, the median age (interquartile range) was 44 (38–48) years. From 106,466 eligible participants, 3,318 were excluded for having incomplete or invalid information on reproductive history. Out of the remaining 103,148 participants with valid information on reproductive history, 16,704 were nulliparous. Among the 86,444 parous women, 11,023 did not give information about lactation history. Thus, our study population was 75,421 parous women.

### 2.2 Parity and mean duration of lactation per child assessment

Details on up to 10 previous pregnancies were available at baseline. Parity was considered as the number or live births and stillbirths and was added for each woman up to current age. For each live birth, women were asked if they had breastfed and for the duration of breastfeeding in months. Total breastfeeding in months was divided by the number of live births to obtain the mean duration of lactation per child. No information on the type of breastfeeding (i.e., whether it was exclusive, predominant, or partial) was available.

### 2.3 Determination of weight change

Weight at 18 years of age and current weight (in kilograms) was self‐reported at enrolment. Weight change was defined as the difference between current weight and weight at 18 years of age. A prior validation study in the MTC showed a correlation coefficient of 0.89 between current self‐reported and objectively measured weight. Sensitivity and specificity of self‐reported obesity were 66.5 and 97.0%, indicating that self‐reported anthropometry is valid for epidemiological purposes in adult Mexican women (Ortiz‐Panozo et al., 2017).

### 2.4 Assessment of covariates

Age, age at first birth, age at menarche, gestational weight gain, smoking (current, past, and never), diet, physical activity, educational level, socio‐economic status (SES), and marital status were derived from the self‐reported baseline questionnaire. The average gestational weight gain per pregnancy was calculated as the summation of the weight gain reported in each pregnancy divided by the total number of pregnancies. Usual diet during the previous year before enrolment was assessed using a semiquantitative 141‐item food frequency questionnaire. The validity of our dietary assessment questionnaire has been previously described (Hernandez‐Avila et al., 1998).

Three major energy‐adjusted, orthogonal‐transformed dietary patterns scores were identified and derived using factorial analysis: “healthy” (based on fruits, vegetables, and legumes); “Western” (pizza, hamburgers, and unprocessed and processed red meats); and “modern Mexican” (corn tortillas, hot peppers, sodas, and Mexican street‐food; Monge et al., 2018). Physical activity was ascertained by frequency of engaging in common recreational activities, from which metabolic equivalent task hours per week were derived based on the amount and intensity of reported physical activity. SES was defined as tertiles of the sum of questions on ownership of seven household assets: phone, car, computer, vacuum cleaner, microwave oven, cell phone, and internet. We summed responses to these questions to create an additive SES score and classified women in tertiles into low, medium, and high. Educational level was categorized into high school, graduate, and postgraduate levels; and marital status was categorized as single, married/civil union, and divorce/widow.

### 2.5 Statistical analysis

#### 2.5.1 Parity and long‐term weight gain

We categorized the number of children into the following: 0, 1, 2, 3, and ≥4 children. To assess if parity was associated with weight change from 18 years, we used several multivariable regression models using weight change as dependent variable. For covariates with missing values (SES [7%], smoking status [3%], educational level [5%], and average gestational weight gain [1.5%]), we imputed values using a multiple imputation method to reflect the uncertainty around the “true” value and produce unbiased estimates assuming the missingness at random. We defined two models a priori with progressive adjustment of potential confounders: Model 1: age + weight at 18 years; Model 2: Model 1 + educational level + SES + age at menarche. Additionally, as an exploratory analysis, we further adjusted for other covariates including current behaviours that although these variables were collected after the main exposures (parity and lactation), they can be considered potential confounders as reflecting behaviours during reproductive ages; Model 3: Model 2 + dietary pattern + physical activity + smoking status. Tests for trend were conducted by using the median weight gain for each category of parity, with the medians modelled as continuous variable.

Sensitivity analyses were carried out. First, we carried out an analysis excluding nulliparous women as they may be a special group with special characteristics and difficult to understand possible confounders. Finally, analyses were carried out without adjusting for weight at 18 years.

#### 2.5.2 Mean duration of lactation per child and long‐term weight gain

After excluding nulliparous women, we categorized the mean duration of lactation per child into the following categories: 0, <3, 3 to <6, 6 to <12, and ≥12 months. We compared the distribution in percentages of categorical variables and mean ± standard deviation for continuous variables according to mean duration of lactation per child category. To assess if the mean duration of lactation per child was associated with weight change from 18 years to current, age we defined three models a priori with progressive adjustment of potential confounders: Model 1: age + weight at 18 years; Model 2: Model 1 + educational level + SES; Model 3: Model 2 + age at menarche + age at first pregnancy + average gestational weight gain per pregnancy + parity. In an exploratory analysis, we further adjusted for current behaviours; Model 4: Model 3 + dietary pattern + physical activity + smoking status. We conducted tests of linear trend across categories of mean duration of lactation per child by assigning the median value for each category and fitting this continuous variable in the models and a quadratic term of this same variable to evaluate the quadratic trend. We also conducted prespecified subgroup analyses by potential effect modifier of median body mass index (BMI) at 18 years (<21/≥21 kg/m^2^) using the multivariable‐adjusted model. The interaction between the continuous term for lactation and BMI at 18 years was examined using the likelihood ratio test (LRT), with a *P*‐value < .05 considered statistically significant.

In addition, we examined the possibly of non‐linear dose–response association between mean duration of lactation per child and total long‐term weight change with restricted cubic splines (Greenland, 1995) with knots at 3, 6, 9, and 12 months of lactation per child distribution. Adjustment was made for age, weight at 18 years, educational level, SES, age at menarche, age at first pregnancy, average gestational weight gain per pregnancy, parity, dietary pattern, physical activity, and smoking status.

Sensitivity analyses were carried out. The same analysis was carried out in uniparous women because in this group, confounding by later births and subsequent lactation would be absent.

All statistical tests were two‐sided and considered statistically significant if *P* < .05. All analyses were conducted using Statistical Analysis System (SAS) software package, Version 9.3 (SAS Institute, Cary, NC).

### 2.6 Ethical considerations

All women gave informed consent at baseline. The study was approved by the Institutional Review Board at the National Institute of Public Health of Mexico (INSP for its Spanish name).

## 3 RESULTS

The median number of children was 2.0 children (interquartile range 1–3). Women with four or more (median 4) children were older, had higher current BMI and lower weight at 18 years, were younger at first pregnancy, and were more likely to be married than nulliparous women. Also, they were less educated but with higher SES than nulliparous women (Table 1). Current behaviours (diet, smoking, and physical activity) were uncorrelated with parity.

**Table 1:** Baseline characteristics of 103,148 women in the Mexican Teachers' Cohort according to parity

Maternal characteristics	Parity				
	0 (*n* = 16,704)	1 (*n* = 15,275)	2 (*n* = 30,150)	3 (*n* = 25,514)	≥4 (*n* = 15,505)
Age (years)	41.4 (8.4)	41.4 (8.0)	43.9 (6.8)	45.7 (6.2)	47.5 (6.2)
Age at first birth (years)	—	28.6 (5.3)	25.8 (4.3)	24.0 (3.9)	22.7 (3.6)
Age at menarche (years)	12.4 (1.5)	12.5 (1.5)	12.5 (1.5)	12.6 (1.5)	12.7 (1.5)
Weight at 18 years (kg)	54.1 (10.1)	53.3 (8.8)	52.3 (8.0)	52.0 (7.6)	52.1 (7.4)
Current weight (kg)	67.3 (13.7)	67.8 (13.0)	67.9 (12.1)	69.3 (12.1)	69.8 (11.9)
Current BMI (kg/m^2^)	26.7 (5.0)	26.9 (4.7)	27.2 (4.4)	27.8 (4.4)	28.1 (4.5)
Gestational weight gain (kg)	—	12.5 (3.8)	17.9 (4.9)	18.4 (5.5)	17.9 (5.5)
Family history of diabetes (%)	53.5	54.3	56.2	57.8	58.4
Dietary pattern (%)					
(Quintile 5)	19.5	18.6	19.9	20.6	20.6
(Quintile 5)	20.9	21.2	20.9	19.9	16.7
Mexican (Quintile 5)	18.7	18.8	18.9	21.4	22.7
Physical activity (MET hr/week)	35.4 (32.7)	33.1 (30.9)	33.8 (31.1)	34.4 (32.1)	35.1 (32.7)
Married (%)	29.6	60.9	82.2	86.4	85.2
Smoking history (%)					
Never	80.9	78.2	78.1	78.3	77.7
Former	10.2	12.6	12.2	12.3	12.9
Current	8.8	9.2	9.7	9.4	9.3
Postgraduate education (%)	22.6	16.9	13.4	12.6	12.6
Socio‐economic status (%)					
Low (Tertile 1)	23.5	25.3	19.1	18.4	20.7
Medium (Tertile 2)	33.9	33.2	28.7	27.4	27.3
High (Tertile 3)	42.6	41.4	52.2	54.1	52.3

*Note.* Values are mean (*SD*) unless otherwise specified. Baseline was defined as enrolment at MTC (2008).

Abbreviation: MET, metabolic equivalent.

In the MTC, 75,421 parous women reported total duration of lactation. Median of total duration of lactation was 12 months (interquartile range 6–21); and median duration per child was 5.0 months (interquartile range 3–8). In this population, women who breastfed for longer periods per child had slightly lower current BMI and weight at 18 years, were more likely to be married, were more likely to be engaged in vigorous physical activity, and were more likely to have a healthy dietary pattern. Also, they were less likely to be smokers than were women with the shortest duration of lactation per child (Table 2).

**Table 2:** Baseline characteristics of 75,421 parous women in the Mexican Teachers' Cohort according to mean duration of lactation per child

Characteristics	Mean duration of lactation per child				
	0 months (*n* = 2,545)	<3 months (*n* = 16,457)	3 to <6 months (*n* = 22,643)	6 to <12 months (*n* = 23,637)	≥12 months (*n* = 10,139)
Age (years)	45.2 (7.1)	44.9 (7.0)	44.8 (6.9)	44.2 (6.9)	43.9 (6.9)
Age at first birth (years)	24.2 (4.3)	24.9 (4.5)	24.8 (4.4)	25.2 (4.5)	25.2 (4.6)
Age at menarche (years)	12.4 (1.4)	12.5 (1.4)	12.5 (1.4)	12.6 (1.5)	12.7 (1.7)
Weight at 18 years (kg)	52.8 (8.5)	52.7 (8.2)	52.3 (7.8)	52.1 (7.5)	52.0 (7.6)
Current weight (kg)	70.5 (13.2)	69.3 (12.8)	68.6 (12.2)	68.2 (11.7)	67.9 (11.8)
Current BMI (kg/m^2^)	28.1 (4.8)	27.6 (4.7)	27.3 (4.5)	27.2 (4.4)	27.4 (4.4)
Gestational weight gain (kg)	17.2 (5.5)	17.0 (5.4)	17.3 (5.4)	17.2 (5.4)	16.7 (5.3)
Family history of diabetes (%)	56.8	56.6	56.9	55.9	56.1
Dietary pattern (%)					
Healthy (Quintile 5)	19.1	19.5	20.8	20.9	20.1
Western (Quintile 5)	23.2	22.7	20.6	18.2	15.1
Modern Mexican (Quintile 5)	22.8	20.6	18.5	18.2	21.5
Physical activity (MET hr/week)	32.8 (30.7)	33.0 (30.6)	34.6 (31.4)	35.6 (32.4)	35.7 (33.1)
Married (%)	77.2	76.0	78.4	79.7	78.7
Smoking history (%)					
Never	72.8	74.8	76.3	80.2	83.4
Former	16.1	13.9	13.3	11.4	9.4
Current	11.1	11.2	10.4	8.3	6.8
Postgraduate education (%)	13.8	13.9	14.3	13.4	12.2
Socio‐economic status (%)					
Low (Tertile 1)	16.9	17.2	16.7	20.7	27.1
Medium (Tertile 2)	28.0	26.9	28.8	29.6	30.5
High (Tertile 3)	55.1	55.8	54.4	49.6	42.4

*Note.* Values are mean (*SD*) unless otherwise specified. Baseline was defined as enrolment at MTC (2008).

Abbreviation: MET, metabolic equivalent.

The overall mean (*SD*) weight change from age 18 years to current age was 16.1 (10.9) kg over an average period of 26 (interquartile range 23–31) years (average 0.62 kg/year). The prevalence of overweight and obesity increased from 8.1% and 1.4% at 18 years to 42.2% and 25.6% at the current age, respectively. Two thirds (66.2%) of women with normal weight at 18 years became overweight or obese at the time of baseline questionnaire (mean age 44 years).

The mean weight gain and the prevalence of overweight and obesity increased with parity. Parous women gained more weight than did nulliparous women, and this weight gain was greater and consistent among categories of parity above that normally age‐related weight gain. After adjustment for age, weight at 18 years, age at menarche, education, and SES, women who had ≥4 children gained 2.81 kg more (95% CI [2.52, 3.10]; *P* for trend < .0001) than had nulliparous women. When lifestyle behaviours was adjusted for (dietary pattern, physical activity, and smoking status), estimates were slightly attenuated, but results remained significant as compared with those of nulliparous women (Table 3).

**Table 3:** Maternal weight change from 18 years to current age (95% CI) in relation to parity in the Mexican Teachers' Cohort

	Parity									*P* trend
	Nulliparous (*n* = 16,704)	1 child (*n* = 15,275)		2 children (*n* = 30,150)		3 children (*n* = 25,514)		≥4 children (*n* = 15,505)		
Weight change (kg)^a^	12.2 (10.3)	15.1 (10.2)		16.4 (10.1)		17.9 (10.4)		18.4 (10.4)		
Model 1	Reference	1.04	[0.77, 1.32]	1.54	[1.30, 1.78]	2.60	[2.36, 2.85]	2.69	[2.41, 2.98]	<.0001
Model 2	Reference	1.08	[0.81, 1.36]	1.61	[1.37, 1.85]	2.72	[2.47, 2.97]	2.81	[2.52, 3.10]	<.0001
Model 3	Reference	1.11	[0.82, 1.42]	1.50	[1.24, 1.76]	2.53	[2.26, 2.80]	2.67	[2.37, 2.98]	<.0001

*Note.* Model 1 = Age + weight at 18 years. Model 2 = Model 1 + educational level + SES + age at menarche. Model 3 = Model 2 + dietary pattern + physical activity + smoking status.

a Mean (*SD*).

When nulliparous women were excluded, compared with those of uniparous women, the adjusted mean (95% CI) weight gain differences were 0.45 [0.17, 0.75], 1.69 [1.37, 2.00], and 2.29 [1.92, 2.65] kg for women with 2, 3, and ≥4 children, respectively (*P* for trend < .001). In this sensitivity analysis, we included a third model that included other pregnancy‐related variables such as age at first pregnancy and the average gestational weight gain per pregnancy that can be considered potential mediator between parity and weight change during reproductive age (Table S1). Also, the analysis carried out without adjusting for weight at 18 years yielded similar results, and long‐term weight gain was associated with parity (Table S2).

**Figure 1:**
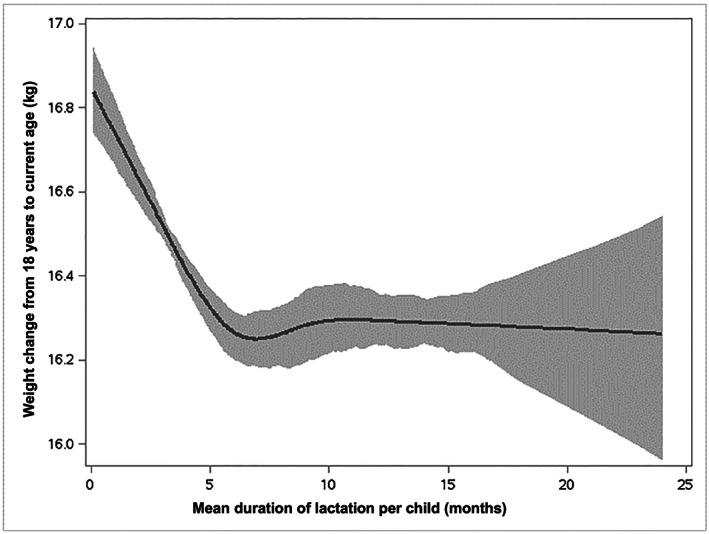
Adjusted dose–response association between mean duration of lactation per child and weight change from 18 years to current age. Data were modelled with restricted cubic splines using generalized least squares regression with 4 knots (3, 6, 9, and 12 months). Grey line plots the predicted weight change from 18 years to current age values with 95% confidence intervals (light grey fill)

The association between mean duration of lactation per child and weight change appeared to be non‐linear. We found that women who breastfed on average 3–6 months per child had a modest but significant lower gain weight (−1.10, 95% CI [−1.58, −0.47 kg], respectively) compared with that of women who did not breastfeed, and that maternal weight change did not differ after six or more months of lactation per child (Table 4). Figure 1 shows the adjusted dose–response association between mean duration of lactation per child and long‐term weight change. In the analysis with restricted cubic splines, we observed that women who breastfed on average 3–6 months per child had a modest but significantly lower gain weight, and after 6 months of lactation per child, the association appeared to plateau (*P* for non‐linearity < .0001). These findings reinforce the interpretation of Table 4, which suggests plateau after 6 months of lactation per child. Results from the sensitivity analysis carried out in uniparous women yielded similar results, and the association between mean duration of lactation per child and long‐term weight change was linear up to 6 months of lactation per child, after which the less weight gain reached a plateau (Table S3).

**Table 4:** Maternal weight change from 18 years to current age (95% CI) in relation to mean lactation per child in the Mexican Teachers' Cohort

	Mean duration of lactation per child									*P* trend	*P* for quadratic trend
	0 months (*n* = 2,545)	<3 months (*n* = 16,457)		3 to <6 months (*n* = 22,643)		6 to <12 months (*n* = 23,637)		≥12 months (*n* = 10,139)			
Weight change (kg)^a^	17.72 (11.10)	16. 77 (11.03)		16.55 (10.57)		16.34 (10.44)		16.26 (10.41)			
Model 1	Reference	−0.89	[−1.39, −0.38]	−1.13	[−1.62, −0.63]	−1.18	[−1.68, −0.68]	−1.19	[−1.72, −0.66]	.0004	.004
Model 2	Reference	−0.87	[−1.38, −0.37]	−1.11	[−1.61, −0.62]	−1.18	[−1.69, −0.68]	−1.21	[−1.74, −0.68]	.002	.0004
Model 3	Reference	−0.81	[−1.33, −0.28]	−1.11	[−1.62, −0.59]	−1.14	[−1.67, −0.63]	−1.10	[−1.55, −0.44]	.006	.005
Model 4	Reference	−0.82	[−1.39, −0.26]	−1.10	[−1.58, −0.47]	−0.91	[−1.47, −0.36]	−0.74	[−1.34, −0.14]	.04	.03

*Note.* Model 1 = Age + weight at 18 years. Model 2 = Model 1 + educational level + SES. Model 3 = Model 2 + age at menarche + age at first pregnancy + average gestational weight gain + parity. Model 4 = Model 3 + dietary pattern + physical activity + smoking status.

a Mean (*SD*).

The test for interaction between lactation and BMI at 18 years was not statistically significant (*P* for interaction = .03).

## 4 DISCUSSION

We found a positive association between parity and weight gain from 18 years to current age even after adjustment for current age, sociodemographic, and other reproductive and lifestyle characteristics. This weight gain may therefore be attributed to pregnancy‐related factors such as post‐partum weight retention. However, in our exploratory analysis, the inclusion of the average gestational weight gain, which was a predictor for weight change, did not modify the associations. Unfortunately, we do not have information of post‐partum weight retention. In addition, we found that women who breastfed 1 to 6 months per child could reduce cumulative weight gain associated with parity without any further association with longer breastfeeding duration.

The mean weight gain (0.62 kg/year) experienced by the studied Mexican women is consistent with the average annual weight gain reported in other studies. Women participants of the Black Women's Health Study during 4 years of follow‐up had a mean gain weight of 1.1 kg/year (Rosenberg et al., 2003), and white women in the CARDIA study during 10 years of follow‐up gained 0.7 kg/year (Lewis et al., 2000). Long‐term weight gain has been associated with parity in other studies. For example, in the Black Women's Health Study, the BMI of women who bore her first child increased 1.1 kg/m^2^ (equivalent to a difference in weight gain of approximately 3.0 kg) more than that of nulliparous women (Rosenberg et al., 2003). In the CARDIA study, an excess weight gain of 3.0 to 6.0 kg was associated with having one or more pregnancies relative to nulliparous women (Gunderson et al., 2004). Moreover, findings from longitudinal studies support the positive association between parity and risk of being overweight or obese after pregnancy (Kim, Stein, & Martorell, 2007; Weng et al., 2004).

We compared weight change in parous women with nulliparous women to account for age‐related weight gain, having found a significant long‐term weight gain in parous women, suggesting that weight gain is related to pregnancy factors. As in other studies (Rooney, Schauberger, & Mathiason, 2005; Rosenberg et al., 2003), we found that weight gain during pregnancies was positively associated with long‐term weight gain during reproductive age (*β* = .31, *P* < .0001). This is particularly important because the main mechanism by which parity is thought to contribute to overweight and obesity in women of reproductive age is through cumulative cycles of excessive gestational weight gain and post‐partum weight retention (Kim et al., 2007).

Regarding the association between mean duration of lactation per child and long‐term weight change, other studies have shown that from 3 to 6 months post‐partum, breastfed women experienced greater weight loss compared with nonlactating women (Jarlenski, Bennett, Bleich, Barry, & Stuart, 2014; Sadurskis, Kabir, Wager, & Forsum, 1988; van Raaij, Schonk, Vermaat‐Miedema, Peek, & Hautvast, 1991). Moreover, data from two randomized trials indicated that greater frequency of breastfeeding from 4 to 6 months post‐partum led to greater post‐partum weight loss (Dewey, Cohen, Brown, & Rivera, 2001). Our data suggest that this weight loss in the post‐partum period persists for several years after childbirth, yielding lower weight gain later in life. This is of critical health importance because even a modest 1% reduction in BMI would substantially reduce the number of obesity‐related illness (Wang, McPherson, Marsh, Gortmaker, & Brown, 2011). Additionally, our study provides novel important information on the dose–response association between mean duration of lactation per child and long‐term weight gain. We observed a plateau on weight after 6 months of lactation per child. This could potentially be explained by the fact that after 6 months of age, the frequency and intensity of lactation decrease as complementary foods are introduced (World Health Organization, 2017). However, we did not have information about the type of breastfeeding to explore this potential explanation to further explore this hypothesis.

Given that breastfeeding also promotes infant health, growth, and development, straightforward strategies such as peer counselling could be useful to improve rates of breastfeeding initiation, duration, and exclusivity. Peer counselling has been identified an effective initiative that can be scaled up in both developed and developing countries, as part of well‐coordinated national breastfeeding promotion or maternal–child health programmes (Chapman, Morel, Anderson, Damio, & Perez‐Escamilla, 2010).

The non‐linear association between mean duration of lactation per child and long‐term weight gain may explain, among other factors, why other studies that have assumed a linear relation have not found an association (Palmer et al., 2015). Other factors that may explain the inconsistency in findings could result from heterogeneity in measures of duration of lactation, duration of follow‐up, low statistical power, retention rates, and differences in characteristics of study population. The observed association between lactation and lower long‐term weight has biological plausibility because lactation may play a central role in mobilizing accumulated fat stores accumulated during pregnancy (due to increased energy expenditures), particularly visceral fat (Stuebe & Rich‐Edwards, 2009), and lactating women are more likely to mobilize fat stores after 3 months post‐partum than are nonlactating women (Stuebe & Rich‐Edwards, 2009). In addition, it has been suggested that lactation could regulate appetite through the adipokines ghrelin and peptide YY, yielding the observed lower weight gain among breastfed women (Stuebe et al., 2011).

Our study had strengths and limitations. Current self‐reported weight has been validated in our cohort, and we had data on potential confounders. In addition, we assessed long‐term intraindividual weight change in reproductive years. When evaluating the association between parity and long‐term weight gain, we used a comparison group of nulliparous women to account for age‐related weight gain. However, data on exclusive, partial, or predominant breastfeeding were not assessed, nor were pregestational and postgestational weight; therefore, we were not able to differentiate between post‐partum weight retention and weight gain in the subsequent period. Also, we assumed that the average gestational weight gain was weight gained in each pregnancy, but it could be possible that the women may begin each pregnancy at a different (probably higher) weight than the previous pregnancy. In addition, we assumed that lifestyle characteristics (e.g.*,* dietary pattern and physical activity) assessed at enrolment and therefore postexposure (parity or lactation) were the same during all reproductive life, including interpregnancy periods. However, given the consistency of the association after adjusting for these postexposure variables, it seems unlikely that a better measurement of these variables during reproductive ages could explain the association that we found. In addition, all participants were teachers, which may increase internal validity but may decrease generalizability to other populations. Finally, although we adjusted for reproductive, behavioural, and sociodemographic characteristics, confounding by unmeasured and unknown factors cannot be ruled out. For example, a high pregestational BMI and metabolic alterations like insulin resistance could negatively affect the initiation and duration of breastfeeding. Therefore, a successful breastfeeding may be a marker for adequate prepregnancy BMI and of a nonaltered metabolism that led to a less weight gain later in life.

In conclusion, our results suggest that parity alters weight‐gain trajectory in women and that women who breastfed could reduce this alteration. Furthermore, our results provide evidence of the need to include breastfeeding counselling and support before, during, and after pregnancy to avoid excessive long‐term weight gain through reproductive life. In addition, our results have policy implications, as our findings inform of efforts to seek to extend maternity leave to a 6‐months period beyond the 14 weeks proposed by the International Labour Organization (2014).

## CONFLICTS OF INTEREST

ML and RL‐R received limited salary support from Bloomberg Philanthropies through an institutional grant to the National Institute of Public Health in Mexico. ML and RL‐R received a nonrestricted investigator‐initiated grant from AstraZeneca. MM, EO‐P, and TGdC declare no conflicts of interest.

## CONTRIBUTIONS

RL‐R and MM designed the study; MM and EO‐P conducted the data analysis; MM drafted the article. All authors contributed to the interpretation of data discussed in the manuscript, revised the manuscript, and approved its final version. RL‐R has primary responsibility for final content.

## Supporting information

Table S1. Maternal weight change from 18 years to current age (95% CI) in relation to parity in the Mexican Teachers’ Cohort (excluding nulliparous women)
Table S2. Maternal weight change from 18 years to current age (95% CI) in relation to parity in the Mexican Teachers’ Cohort (without adjusting for weight at 18 years)
Table S3. Maternal weight change from 18 years to current age (95% CI) in relation to mean duration of lactation per child in women with just 1 pregnancy in the Mexican Teachers’ Cohort
