# Supplementary material for: Parity, lactation, and long‐term weight change in Mexican women
Source: Matern Child Nutr. 2020 Mar 24;16(3):e12988. doi: 10.1111/mcn.12988 (PMC7296806; doi:10.1111/mcn.12988)
Supplement: Supplementary file 1 — Table S1. Maternal weight change from 18 years to current age (95% CI) in relation to parity in the Mexican Teachers’ Cohort (excluding nulliparous women) Table S2. Maternal weight change from 18 years to current age (95% CI) in relation to parity in the Mexican Teachers’ Cohort (without adjusting for weight at 18 years) Table S3. Maternal weight change from 18 years to current age (95% CI) in relation to mean duration of lactation per child in women with just 1 pregnancy in the Mexican Teachers’ Cohort [file MCN-16-e12988-s001.docx]

**Supplementary Material**

**Supplementary Table 1. Maternal weight change from 18 years to current age (95% CI) in relation to parity in the**

**Mexican Teachers’ Cohort (excluding nulliparous women)**

|  | **Parity** | | | | | | |  | |
| --- | --- | --- | --- | --- | --- | --- | --- | --- | --- |
|  | **1 child**  **(n=15,275)** | **2 children**  **(n=30,150)** | | **3 children**  **(n=25,514)** | | **≥4 children**  **(n=15,505)** | | | *P* for trend |
| Mean weight change (kg)^a^ | 15.1 (10.2) | 16.4 (10.1) | | 17.9 (10.4) | | 18.4 (10.4) | | |  |
| Model 1 | Reference | 1.04 | (0.84, 1.23) | 2.10 | (1.89, 2.31) | 2.18 | (1.94, 2.43) | | <0.0001 |
| Model 2 | Reference | 1.04 | (0.84, 1.23) | 2.10 | (1.89, 2.31) | 2.18 | (1.94, 2.43) | | <0.0001 |
| Model 3 | Reference | 0.61 | (0.34, 0.89) | 1.94 | (1.64, 2.23) | 2.51 | (2.17, 2.86) | | <0.0001 |
| Model 4 | Reference | 0.45 | (0.17, 0.75) | 1.69 | (1.37, 2.00) | 2.29 | (1.92, 2.65) | | <0.0001 |

^a^Mean (SD).

Model 1= Age + weight at 18 years.

Model 2= Model 1 + educational level + SES.

Model 3= Model 2 + age at menarche + age at first pregnancy + average gestational weight gain.

Model 4= Model 3 + dietary pattern + physical activity + smoking status.

**Supplementary Table 2. Maternal weight change from 18 years to current age (95% CI) in relation to parity in the**

**Mexican Teachers’ Cohort (without adjusting for weight at 18 years)**

|  | **Parity** | | | | | | |  |
| --- | --- | --- | --- | --- | --- | --- | --- | --- |
|  | **1 child**  **(n=15,275)** | **2 children**  **(n=30,150)** | | **3 children**  **(n=25,514)** | | **≥4 children**  **(n=15,505)** | | *P* for trend |
| Mean weight change (kg)^a^ | 15.1 (10.2) | 16.4 (10.1) | | 17.9 (10.4) | | 18.4 (10.4) | |  |
| Model 1 | Reference | 1.25 | (1.06, 1.46) | 2.32 | (2.11, 2.54) | 2.35 | (2.10, 2.60) | <0.0001 |
| Model 2 | Reference | 1.27 | (1.07, 1.47) | 2.32 | (2.11, 2.54) | 2.34 | (2.09, 2.59) | <0.0001 |
| Model 3 | Reference | 0.74 | 0.47, 1.02) | 2.05 | (1.75, 2.35) | 2.52 | (2.17, 2.86) | <0.0001 |
| Model 4 | Reference | 0.59 | 0.25, 0.88) | 1.80 | (1.48, 2.12) | 2.28 | (1.91, 2.66) | <0.0001 |

^a^Mean (SD).

Model 1= Age.

Model 2= Model 1 + educational level + SES.

Model 3= Model 2 + age at menarche + age at first pregnancy + average gestational weight gain.

Model 4= Model 3 + dietary pattern + physical activity + smoking status.

**Supplementary Table 3. Maternal weight change from 18 years to current age (95% CI) in relation to mean duration of lactation per child in women with just 1 pregnancy in the Mexican Teachers’ Cohort**

|  | **Mean duration of lactation per child** | | | | | | | | | *P* for trend | *P* for quadratic trend |
| --- | --- | --- | --- | --- | --- | --- | --- | --- | --- | --- | --- |
|  | **0 months**  **(n=1,547)** | **<3 months**  **(n=977)** | | **3 - <6 months**  **(n=2,917)** | | **6 - <12 months**  **(n=3,682)** | | **≥12 months**  **(n=2,141)** | |  |  |
| Weight change (kg)^a^ | 15.47 (10.73) | 14.95 (11.38) | | 14.85 (10.76) | | 14.20 (10.14) | | 14.74 (10.30) | |  |  |
| Model 1 | Reference | -0.34 | (-1.32, 0.64) | -0.87 | (-1.62, -0.12) | -1.19 | (-1.92, -0.42) | -0.80 | (-1.60, -0.10) | 0.001 | 0.002 |
| Model 2 | Reference | -0.35 | (-1.33, 0.62) | -0.86 | (-1.61, -0.11) | -1.19 | (-1.92, -0.42) | -0.81 | (-1.62, -0.10) | 0.001 | 0.002 |
| Model 3 | Reference | -0.41 | (-1.41, 0.60) | -0.86 | (-1.61, -0.10) | -1.07 | (-1.83, -0.32) | -0.65 | (-1.41, 0.18) | 0.004 | 0.006 |
| Model 4 | Reference | -0.25 | (-1.34, 0.84) | -0.64 | (-1.48, 0.20) | -0.70 | (-1.52, 0.12) | -0.26 | (-1.17, 0.65) | 0.08 | 0.06 |

^a^Mean (SD).

Model 1= Age + weight at 18 years.

Model 2= Model 1 + educational level + SES.

Model 3= Model 2 + age at menarche + age at first pregnancy + average gestational weight gain.

Model 4= Model 3 + dietary pattern + physical activity + smoking status.
